# Supplementary material for: AmAtg2B-Mediated Lipophagy Regulates Lipolysis of Pupae in Apis mellifera
Source: Int J Mol Sci. 2023 Jan 20;24(3):2096. doi: 10.3390/ijms24032096 (PMC9916532; doi:10.3390/ijms24032096)
Supplement: Supplementary file 1 [file ijms-24-02096-s001.zip › ijms-2074699-supplementary.pdf]

Materials and methods

1. RNA isolation，cDNA synthesis, and fluorescent real-time quantitative PCR (RT-qPCR) analyses

Total RNA of samples was extracted using the RNAiso Plus (TaKaRa, Dalian, China) according to the manufacturer's instructions.

The first-strand cDNA of mRNA and mature miRNA were synthesized for fluorescent real-time quantitative polymerase chain reaction (qPCR) from total RNA using *Evo M-MLV* RT Premix for qPCR (Accurate Biology, China) and miRNA 1st Strand cDNA Synthesis kit (Accurate Biology, China), respectively.

Fluorescent real-time quantitative PCR (qRT-PCR) was carried out to determine the expression levels of mRNA by using the SYBR® Green Premix *Pro Taq* HS qPCR Kit (Rox Plus) (Accurate Biology, China). The reaction condition of RT-qPCR was a two-step PCR reaction program (Step 1: 95°C for 30s, Step 2:40 cycles of 95°C for 5 s and 60°C for 34 s). Relative expression of mRNA was analyzed with the CFX Manager software (version 1.1), using the 2 (-delta delta CT) method [1] and amplification of the *Actin* transcript (Gene ID: LOC108003299) [2]. Six biological replicas were set for each treatment group, and the PCR reactions were performed in triplicate.

2. LC/MS non-targeted lipidome analysis

Sample preparation

Using homogenizer to break up the samples, 300 μL MeOH was added and vortexed

for 1min (4°C, 2,000 rpm), followed by sonication in an ice water bath for 1min; 1,000 μL MTBE was added and vortexed for 1min (4°C, 2,000 rpm), then left at 4°C for 30 min; then added 300μL water to vortex for 1min (4°C, 2000 rpm), and let stand at 4℃ for 10 min; The samples were centrifuged at 12,000 rpm for 15 min at 4°C, 1,500 μL of supernatant was placed in EP tube and dried under vacuum; 100μL of solution (DCM:MeOH:H2O=60:30:4.5) was added for redissolution, vortexed for 30 s, and sonicated in ice water bath for 5 min. Finally, the samples were centrifuged at 12,000 rpm for 10 min at 4°C. 20 μL of the supernatant was put into the injection bottle for for subsequent UPLC-MS/MS analysis. Another 10 μL supernatant of all samples was mixed into QC samples for machine detection. QC samples were prepared by mixing aliquot of the all samples to be a pooled sample.

Thermo Q-Exactive /Dionex U3000 UHPLC

A Dionex Ultimate 3000 RS UHPLC fitted with Q-Exactive quadrupole-Orbitrap mass spectrometer equipped with heated electrospray ionization (ESI) source (Thermo Fisher Scientific, Waltham, MA, USA) was used to analyze the metabolic profiling in both ESI positive and ESI negative ion modes. An ACQUITY UPLC BEH C18 column (1.7μm, 2.1×100mm) were employed in both positive and negative modes. The binary gradient elution system consisted of (A) acetonitrile:water (60:40, v:v, containing 10mmol/L ammonium formate) and (B) acetonitrile: isopropanol (10:90, v:v, containing 10mmol/L ammonium formate) and separation was achieved using the following gradient:0 min, 5% B; 0.5 min, 5% B; 2 min, 43% B; 32.1 min, 52% B; 8.5 min, 53% B;

8.6 min,75% B;114 min, 90% B, 14.5in, 100% B, 15.5min, 100% B, 15.7min, 5% B and 18min, 5%B. The flow rate was 0.4mL/min and column temperature was 60℃. All the samples were kept at 4°C during the analysis. The injection volume was 5μL.

Positive: Heater Temp 350°C, Sheath Gas Flow rate 50 arb, Aux Gas Flow Rate15 arb, Sweep Gas Flow Rate 1arb, spray voltage 3.8KV, Capillary Temp 320°C, S-Lens RF Level 75%. MS1 scan ranges: 135-2000.

Negative: Heater Temp 350°C, Sheath Gas Flow rate 50arb, Aux Gas Flow Rate 15arb, Sweep Gas Flow Rate 1arb, spray voltage 3.0KV, Capillary Temp 320°C, S-Lens RF Level 75%. MS1 scan ranges: 135-2000.

The QCs were injected at regular intervals throughout the analytical run to provide a set of data from which repeatability can be assessed.

Data Preprocessing and Statistical Analysis

Peak extraction: The original Q Exactive LC-MS/MS data in .raw format were processed by software Lipid Search for MSn and the exact mass-to-charge ratio (m/z) of parent ions.

Identification: The molecular structure of lipids and the additive mode of its positive and negative ions were identified according to the parent ions and multi-stage mass spectrometry data of each individual sample.

Peak alignment: The results were aligned according to a certain retention time range and combined into a single report to sort out the original data matrix.

In each sample, all peak signals were normalized (that is, the signal intensity of each

peak is converted to the relative intensity in the spectrum, and then multiplied by 10,000). The extracted data were then further processed by removing any peaks with a missing value (ion intensity = 0) in more than 50% in groups and by replacing the zero value by half of the minimum value. A data matrix was combined from the positive and negative ion data.

The matrix was imported in R to carry out Principle Component Analysis (PCA) to observe the overall distribution among the samples and the stability of the whole analysis process. Orthogonal Partial Least-Squares-Discriminant Analysis (OPLS-DA) and Partial Least-Squares-Discriminant Analysis (PLS-DA) were utilized to distinguish the metabolites that differ between groups. To prevent overfitting, 7-fold cross-validation and 200 Response Permutation Testing (RPT) were used to evaluate the quality of the model.

Variable Importance of Projection (VIP) values obtained from the OPLS-DA model were used to rank the overall contribution of each variable to group discrimination. A two-tailed Student's T-test was further used to verify whether the metabolites of difference between groups were significant. Differential metabolites were selected with VIP values greater than 1.0 and *P*-values less than 0.05.

LC-MS /MS protein identification

Enzymolysis with pancreatic enzymes

Transfered the cut protein band into 1.5mL centrifuge tube and rinse twice with ultra-pure water;

Added decolorization solution and decolorize for 30 min (subject to complete

decolorization); discarded decolorization solution, added dehydration solution 1 (50% ACN in water), and dehydrate for 30min; Sucked out dehydration solution 1 (50% ACN in water), add dehydration solution 2 (100% ACN), dehydrate for 30min, vacuum freeze dry; added 50μL of reducing solution to the lyophilized glue block in a warm bath at 55°C for 1h; Sucked out the liquid, cool to room temperature, add 50μL of reducing solution 2 (10mM DTT/25mM NH4HCO3), and placed in the dark for 30 min; Sucked out the liquid and added the imbibition solution for 10min; Sucked out the imbibition solution, added dehydration solution 1 (50% ACN in water), and dehydrate for 30 min; Sucked out dehydration solution 1, add dehydration solution 2, and dehydrated for 30min; Sucked out dehydration solution 2, add 10μl of enzymatic working solution (0.02μg/μL trypsin in 25mM NH4HCO3), imbibition for 30 min; added 20μL of enzymatic coating solution, and enzymatic hydrolysis for 16 h in 37°C water bath; After enzymatic hydrolysis, the supernatant was transferred to a new centrifuge tube; Added 50μL peptide extract to the remaining glue, water bath at 37°C for 20 min, centrifugation at 5,000g for 5 min, merge the supernatant, repeat the above operation once again, and wave dry before desalting.

Desalinisation the eluted peptides

1. Prepare C18 membrane filled column;

2. Re-dissolve the dried peptide sample in Nano-HPLC Buffer A (0.1% formic acid in water);

3. Activation: Centrifugation with 40μL methanol once, discard the liquid at the

bottom of EP tube, repeat twice;

4. Equilibrium: 40μL Nano-HPLC Buffer A (0.1% formic acid in water) was centrifuged through the column once, the liquid at the bottom of the EP tube was discarded, and repeated twice;

5. Solid peptide: the peptide sample was centrifuged through the column once, and the liquid at the bottom of the EP tube was centrifuged through the column once more;

6. Desalting: 40μL Nano-HPLC Buffer A (0.1% formic acid in water) was centrifuged through the column once, the liquid at the bottom of the EP tube was discarded, and repeated twice;

7. Elution: Replace the EP tube with a new EP tube, centrifuged 40μL of elution phase Buffer B (0.1% formic acid/50% ACN in water) through the column once, collected the liquid at the bottom of the EP tube, and repeated once.

8. After desalting, 80μL of elution phase Buffer B (0.1% formic acid/50% ACN in water) containing peptide samples was dried by waving.

Thermo Q-EXACTIVE /Dionex U3000 UHPLC

The nano-HPLC liquid phase system Easy-NLC1200 was used for separation, the dried polypeptide samples were first re-dissolved in Nano-HPLC Buffer A, liquid A was 0.1% formic acid-aqueous solution, and liquid B was 0.1% formic acid-acetonitrile solution. 100μm×20mm (RP-C18, Thermo Inc.) with 100% liquid A equilibrium. The samples were then loaded by an automatic sampler and adsorbed to a Trap column, and separated on an Analysis column, 75μm×150mm (rp-c18, Thermo Inc.) at a flow rate of

300nL/min. The samples were cleaned by mobile phase gradient with blank solvent for 30min. The hydrolysates were separated by capillary high performance liquid chromatography and analyzed by Q-Exactive plis mass spectrometry (Thermo Scientific).

The scanning range of parent ions was corrected by standard correction fluid at 300-1600 m/z, and the scanning mode of mass spectrometry was Data Dependent Aqcuisition (DDA). The 20 strongest fragment profiles (MS2 Scan) are collected after each full scan. Fragmentation was performed using high-energy collision dissociation (HCD, high energy) with an NCE energy of 28 and dynamic removal time of 25 s. The resolution of MS1 was 45000 at M/Z 200, the AGC target was 1E6, and the maximum injection time was 50 ms. The resolution of MS2 was 15000, the AGC target was 1E5, and the maximum injection time was 50 ms.

Database search

MS/MS spectra were searched using the MaxQuant against the uniprot-*Apis mellifera* (Honeybee) [7460] proteome UP000005203 database. Search database specific parameters are set as follows: Fixed modifications：Carbamidomethyl(C); Variable modification：Oxidation（M）and Acetyl（Protein N-term）; digestion: trypsin; First search peptide tolerance: 20ppm; Main search peptide tolerance: 4.5 ppm; Max Missed: 2.

Table S1. The implementation details of the experimental items.

Experiment items	Groups	First injection		Second injection		Sampling time,sampling quantity and use for
		Time	Substances and doses	Time	Substances and doses	

Autophagy inhibition experiment	CK	PP	PBS, 0.5μL	-	-	Six honey bees of each group were sampled at stage PP, Pw, Pb for the enzyme activity assay; Six honey bees of each group were sampled at stage Pw for the western blotting; Six honey bees of each group were sampled at stage Pb for the qRT-PCR analysis and the LC/MS non-targeted lipidome analysis.
	3-MA	PP	10mM 3-MA, 0.5μL	-	-	
	CQ	PP	5mM CQ, 0.5μL	-	-	
Atg2B Neutralization experiment	CK	Pw	-	-	-	All samples were sampled 24h after injection and Six honey bees of each group were sampled at stage Pw for the western blotting, LC-MS /MS protein identification and LC/MS non-targeted lipidome analysis.
	Rabbit-IgG	Pw	4μg Rabbit-IgG, 0.8μL	-	-	
	Anti-Atg2B	Pw	4μg Rabbit-Anti-Atg2B, 0.8μL	-	-	
Rescue experiment	CK	Pw	-	-	-	60 pupae of each group were used for calculating the proportion of pink eyes and eclosion rate.
	Rabbit-IgG	Pw	4μg Rabbit-IgG, 0.8μL	-	-	
	Anti-Atg2B	Pw	4μg Rabbit-Anti-Atg2B, 0.8μL	48h after Pw	PBS, 0.8μL	
	Anti-Atg2B +PC	Pw	4μg Rabbit-Anti-Atg2B, 0.8μL	48h after Pw	5 mg/mL PC, 0.8μL	
	Anti-Atg2B +C17iso	Pw	4μg Rabbit-Anti-Atg2B, 0.8μL	48h after Pw	5 mg/mL C17iso, 0.8μL	

Table S2. PCR primers in this study.

Target gene	Primer sequences (5′-3′)	Description	GenBank number
β-actin	F: CCGTGATTTGACTGACTACCT	Standard control primer, forward	NM_001185145
	R: AGTTGCCATTTCCTGTTC	Standard control primer, reverse	
LC3	F:TGCACAAAGGGTTGCAGATGT	Primer for mRNA RT-qPCR, forward	XM_395337.7
	R: TCTGCCACTGTTAAATAATCCGGT	Primer for mRNA RT-qPCR, reverse	
Atg2B	F:CTTTCAGAGTGGCAGTGGGC	Primer for mRNA RT-qPCR, forward	XM_026441020.1
	R:CCTGGCGCAGAATATCCAGC	Primer for mRNA RT-qPCR, reverse	
Atg9A	F: AAGTCACGGGAGTTGGAGATGTTTG	Primer for mRNA RT-qPCR, forward	XM_016913672.2
	R: GTTGCTGTCTGCCACATTGGATTTC	Primer for mRNA RT-qPCR, reverse	
	F:GAGGCAACTGGACCAGCTATCATG	Primer for mRNA RT-qPCR, forward	XM_003250851.4

DFCP1	R:GGTTGACTGCACTGACATTTCACG	Primer for mRNA RT-qPCR, reverse	
Lsd2	F: GCTCGCAAAATACCAAGTGTCAAT	Primer for mRNA RT-qPCR, forward	XM_026442713.1
	R: TTCCACAGTGTTAAATGCCCAATG	Primer for mRNA RT-qPCR, reverse	
R7	F: AGAAGCTCCTGGGCAAGAAGAATTG	Primer for mRNA RT-qPCR, forward	XM_006561384.3
	R:TAGGTGCTTGGGCTTGACAATAACC	Primer for mRNA RT-qPCR, reverse	
R10	F:ACACCTAAGGCAACACCTCATCAAC	Primer for mRNA RT-qPCR, forward	XM_006569530.3
	R: GCGGAAGTGTACGTGGAAGATCATC	Primer for mRNA RT-qPCR, reverse	
R18	F:TGCAAAGCACAGTTGGAATGGA	Primer for mRNA RT-qPCR, forward	XM_016917414.2
	R:AGCACCTTGGCCATCTCTGT	Primer for mRNA RT-qPCR, reverse	
	F:AATGCTTGCTATGGAGGTACTGCTG	Primer for mRNA RT-qPCR, forward	

Hmgs1			
	R:CCACCAGTTGGTCTAGCACTTCC	Primer for mRNA RT-qPCR, reverse	XM_026440853.1
	F:AATGCTTGCTATGGAGGTACTGCTG	Primer for mRNA RT-qPCR, forward	
Hmgs2			
	R:CCACCAGTTGGTCTAGCACTTCC	Primer for mRNA RT-qPCR, reverse	XM_397202.7
	F:TGGACCATCCGAAAGAGCAATGTG	Primer for mRNA RT-qPCR, forward	
Acc			
	R:TCTGAACCTGACCAGGGAAGTGTAG	Primer for mRNA RT-qPCR, reverse	XM_006565175.3
	F: TGGTTGTCTAGTCCTCAGGATGT	Primer for mRNA RT-qPCR, forward	
Lipid1			
	R: TTAGGAGCGTCCATAGCCCA	Primer for mRNA RT-qPCR, reverse	XM_006564374.3
	F:TTAATGATCGGCCTTGCACCTGTAG	Primer for mRNA RT-qPCR, forward	
Lipid3			
	R: CACCTATCCTCACGCCCATGTATG	Primer for mRNA RT-qPCR, reverse	XM_006558606.3
	F:TTGGCATAGGACAAATGGTGGCTAC	Primer for mRNA RT-qPCR, forward	

Sut1	R: ACACGTTGCTCGTTCTAGGTTAGG	Primer for mRNA RT-qPCR, reverse	XM_006568616.3
	F:GAAATCCACAGGCTCCAGAAGGTAC	Primer for mRNA RT-qPCR, forward	
Glut	R: TAGACATTCCCCAGCATCCAAAACG	Primer for mRNA RT-qPCR, reverse	XM_026441909.1
	F:ATGTAGAGGACGTTGCAGCAGTTAC	Primer for mRNA RT-qPCR, forward	
Brus	R:CTTTCGCCGCTTTCTTGACAACAC	Primer for mRNA RT-qPCR, reverse	NM_001098234.1
	F: GAGCACGAAACCTATGGCGC	Primer for mRNA RT-qPCR, forward	
NPF	R: CTTTCAATCGAGTCTAACACGTGACA	Primer for mRNA RT-qPCR, reverse	NM_001167720.1
	F: ACATGCATCGTAAGCTTCGACCAAG	Primer for mRNA RT-qPCR, forward	
AKH	R: TCGACAACTCCGATCCTTTGACTTC	Primer for mRNA RT-qPCR, reverse	XM_006563546.3
AKHR	F:AGAAGTCAAAGGATCGGAGTTGTCG	Primer for mRNA RT-qPCR, forward	

	R: GCAATTGTTCCACTGAAGGTTTGGC	Primer for mRNA RT-qPCR, reverse	NM_001040264.1
HSL	F: TGTCGATGTATCTCAACGTGCTGAC	Primer for mRNA RT-qPCR, forward	
	R: AGGCAACTTCCGTGCAATATCAGTC	Primer for mRNA RT-qPCR, reverse	XM_397143.7
FOXO	F:CTTCTTATTACGAATCCCA	Primer for mRNA RT-qPCR, forward	
	R:ATTGACCCATCATAGTGC	Primer for mRNA RT-qPCR, reverse	
InR	F:CCGACCGAGAGCAAGTGTTACG	Primer for mRNA RT-qPCR, forward	
	R: TCAGACGATTCATCCTCACGCATTC	Primer for mRNA RT-qPCR, reverse	XM_026443028.1
CHK	F:GCTGCGACAAAATACACGGAAACC	Primer for mRNA RT-qPCR, forward	
	R:TCCGCTGCCGTATAATCATATTGCC	Primer for mRNA RT-qPCR, reverse	XM_026445875.1
Etnk	F:GGATCAAGTATGCAAAGAACCTCA	Primer for mRNA RT-qPCR, forward	

			XM_006559053.3
	R:AAAGGCCATGTTGGTCTAATTTTCT	Primer for mRNA RT-qPCR, reverse	
	F:AAGCATTATCTCCTCCTGCAAGTCC	Primer for mRNA RT-qPCR, forward	
Pcyt1	R:CTGCGAATATTCAAAACGGCCAGTC	Primer for mRNA RT-qPCR, reverse	XM_026441017.1
	F:AGCTCGAAGTCCTTGGACAG	Primer for mRNA RT-qPCR, forward	
Pcyt2	R:TGTTTCCAGGTTGTGGACTT	Primer for mRNA RT-qPCR, reverse	XM_624316.5
	F:AGATTCGTTGCTCTGTGTCAGTTGG	Primer for mRNA RT-qPCR, forward	
Ptdss	R:CTTGCCACAACAAATGGATGAGAGG	Primer for mRNA RT-qPCR, reverse	XM_016914836.2

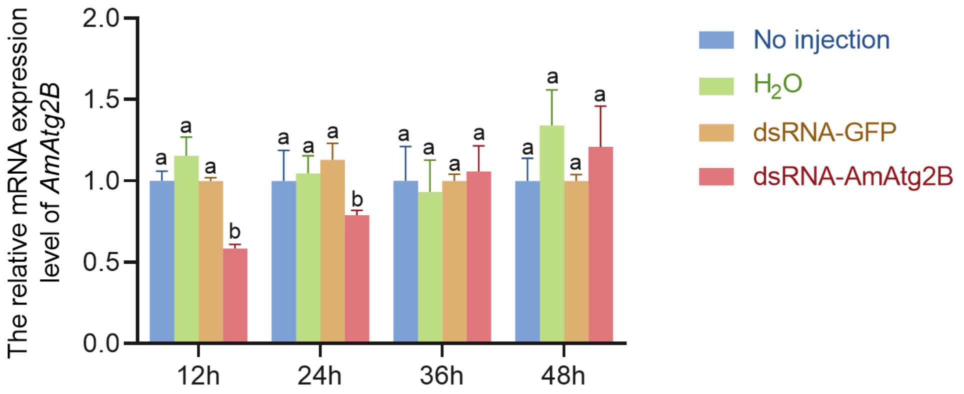

Figure S1. qRT-PCR analysis of expression of *AmAtg2B in* pupae after the injection of dsRNA-*AmAtg2B*. After injectingds RNA-*AmAtg2B* into the hemolymph of Pw, the consecutive expression of A*mAtg2B* up to 48 hours were detected at a interval of 12h by qRT-PCR. *AmAtg2B* showed low expression within 24 hours. Note: Data are shown as mean ± SEM and the different letters marked on the bar chart represent significant differences ($p<0.05$).

References

1. Livak, K.J., and T.D. Schmittgen. Analysis of relative gene expression data using real-time quantitative PCR and the 2(-Delta Delta C(T)) Method. Methods (San Diego, Calif.), 2001, 25(4), 402–408. https://doi.org/10.1006/meth.2001.1262

2. Guidugli, K.R., A.M. Nascimento, G.V. Amdam, A.R. Barchuk, S. Omholt, Z.L. Simões, and K. Hartfelder. Vitellogenin regulates hormonal dynamics in the worker caste of a eusocial insect. FEBS letters, 2005, 579(22), 4961–4965. https://doi.org/10.1016/j.febslet.2005.07.085
